# Co-Occurrence of Beauvericin and Fumonisin Producing Ability of *Fusarium* Strains Isolated from Crop Plants in Hungary

**DOI:** 10.1007/s00284-025-04243-9

**Published:** 2025-05-23

**Authors:** Ákos Suhajda, Mohammed Al-Nussairawi, Ines Amara, Csilla Sörös, Rita Tömösközi-Farkas, Balázs Kriszt, Milán Farkas, Mátyás Cserháti

**Affiliations:** 1 Department of Environmental Safety, Institute of Aquaculture and Environmental Safety, Hungarian University of Agriculture and Life Sciences, Gödöllő, Hungary; 2 Department of Molecular Ecology, Institute of Aquaculture and Environmental Safety, Hungarian University of Agriculture and Life Sciences, Gödöllő, Hungary; 3 Department of Clinical and Laboratory Sciences, College of Pharmacy, University of Misan, Misan, Iraq; 4 Department of Food Chemistry and Analytics, Institute of Food Science and Technology, Hungarian University of Agriculture and Life Sciences, Budapest, Hungary

**Keywords:** Beauvericin, Fumonisin, Fusarium, Emerging mycotoxin

## Abstract

Beauvericin (BEA) is an emerging mycotoxin with wide-ranging bioactivity (antimicrobial and insecticide), making it a potential target for drug and pesticide development. BEA primarily produced by *Beauveria*, *Isaria,* and *Fusarium* species. The BEA-producing abilities of a collection of 100 *Fusarium* strains isolated from maize were tested using a gene-specific primer (Beas_1, Beas_2) by PCR. Among all, 23 were found to have the beauvericin synthetase (BEAS) gene sequence, which is responsible for the production of BEA. *Fusarium proliferatum* (6) and *F. verticillioides* (14) strains were producing the highest BEA concentrations. The toxin-producing ability of the strains was investigated in small bioreactors. Parallel with BEA, the most frequent *Fusarium* toxins such as deoxynivalenol (DON), T2, HT-2, zearalenone (ZEA), fumonisin B1 (FB1), and fumonisin B2 (FB2) were also measured. Only FB1 and FB2 were observed above the detection limit, the coexistence of the FBs and BEA was measured in high concentration. In all BEA-producing strains, the FBs production could be detected. The highest BEA concentration was 3131 mg/kg, and the highest FB1 and FB2 concentrations were 4393 mg/kg and 1390 mg/kg, respectively. In the present study, the gene sequences responsible for the production of BEA in *F. verticilloides* isolates have not only been detected but also demonstrated with UHPLC-ESI-MS/MS to be capable of biosynthesis. From the phylogenic analysis of the BEAS gene sequences, the assumption could be made that the ability to produce BEA was conferred via horizontal gene transfer.

**Supplementary Information:**

The online version contains supplementary material available at 10.1007/s00284-025-04243-9.

## Introduction

Fusariums are one of the most important and well-known phyto-pathogenic and mycotoxin-producing genera, responsible for the production of many major and minor mycotoxin classes. The toxicity of fusariotoxins varies considerably, trichothecenes, fumonisins, and zearalenones are the most hazardous compounds among mycotoxins, not just due to their adverse biological properties, but also because of their very high frequency of occurrence. Mycotoxins can have high thermal and chemical stability and can be present as contaminants throughout the food chain, and many of them can cause both acute and chronic illnesses. Therefore, it is important to monitor their presence continuously in food and feed, as well as to consider and to limit their presence in certain products [1]. Besides toxins that are dangerous from the food safety point of view, there are plenty of non-toxic secondary metabolites or even toxins with lower toxicity which counterweighted with favorable characteristics in certain respects [2]. Although BEA, which belongs to the enniatin antibiotic group, has frequently been isolated in food and feed commodities, it does not appear to pose a significant risk to human health based on acute toxicological studies [3]. Instead, beauvericin has shown many promising beneficial properties for humankind, allowing it to have potential use in agriculture (pesticides) and medicine [4]. Beauvericin (BEA) is a secondary fungal metabolite from the cyclodepsipeptide group, which is produced by different fungal species primarily belonging to *Beauveria*, *Isaria,* and *Fusarium* genera [5]. Although it is considered as an emerging mycotoxin, its biosynthesis was first described in *Beauveria bassiana* by Hamill et al. [6]. Chemically, BEA is a cyclic hexa-depsipeptide that contains three hydroxy-isovaleryl and three N-methyl-phenylalanyl groups in an alternating sequence [4]. The amino acid subunits are linked with peptide and intramolecular ester (lactone) bonds forming a cyclic depsipeptide. The chemical characteristics of BEA explain its anti-inflammatory, anticancer, antibiotic, antiviral, and insecticide attributes, in addition to the positive properties BEA has also shown cytotoxic effects on many cell lines [4, 7]. Additionally, other studies have demonstrated that BEA shows a potentiating effect with other bioactive compounds, thereby broadening the possibilities of its potential applications. According to Shekhar-Guturja et al. [8], BEA has been demonstrated to enhance the efficacy of azole fungicide against azole-resistant clinical isolates of *Candida glabrata* and *Candida albicans* by inhibiting the function of ABC transporters, which are contributes to the multidrug efflux pump effect. A further study by Al Khoury et al. [9] demonstrated that BEA was capable of overcoming ABC transporter-mediated resistance to miltefosine in *Leishmania tropica*. The potentiation of other compounds may also prove disadvantageous. Zouaoui et al. [10] suggest that the simultaneous presence of low concentration of BEA in combination with patulin (PAT) and sterigmatocystin (STE) may induce higher toxicity than PAT or STE individually, or their combination without BEA.

The EFSA Panel on Contaminants in the Food Chain (2014) judged acute exposure to BEA to be non-concerning for human and livestock health [3]. On the other hand, it has been shown that BEA can be cytotoxic in different cell lines depending on the exposure time and toxicological assay [7]. Nevertheless, data about the toxicity and risk assessment of BEA in humans is still sparsely described. As a result, it is still difficult to determine a complete risk assessment for BEA. In addition to its toxicological concerns, the distribution of BEA production appears to be influenced by geographical and genetic factors. There is no unified regulation on systematic monitoring of BEA contamination in the European Union, so the occurrence of this compound is poorly documented and currently this poses a significant challenge to estimating the potential presence of BEA in a broader geographical context, however, according to the World Mycotoxin Survey of 2023, which analyzed 4601 food and feed commodities from 78 countries, BEA was the second most frequently detected mycotoxin (67%, average concentration 49 ppb, maximum concentration 5910 ppb) [11].

FB1 has been classified as a possible human carcinogen and has also been demonstrated to induce nephrotoxic, hepatotoxic, and neurotoxic effects in animals. In the case of FB1 and FB2, there is a high human health risk, and the limit values are the following: FBs on animals, the European Union (EU) defined a recommendation of a maximum of 5 mg FBs (B1 + B2)/kg for complete feed for swine and 1 µg FBs/kg body weight per day as the tolerable daily intake for humans [12]. Due to the high-risk level, usually almost every grain is evaluated for FBs. The significance of FB1 is further supported by the fact that it has been detected in maize samples from around the globe, with levels reaching up to even 300 mg/kg concentrations [13]. According to the results of the 2023 World Mycotoxin Survey [11], very high levels of FBs contamination were detected in the Sub-Saharan region (68%), Middle East and North-African region (94%), in South Africa (54%), in South-East Asia (94%), in Central America (78%), and in South-East Asia (94%).

However, mycotoxins are present in mixed forms in the majority of cases, consequently, strains of *Fusarium* that produce BEA may also produce other *Fusarium* toxins, including T2, DON, FB1, and ZEA, which are due to their frequency of occurrence and their toxicological properties are among the most dangerous mycotoxins and under food and feed safety regulations [14].

Due to its potentiating effect and its beneficial properties, BEA could make interest in pesticide and drug development, therefore, our objective was to investigate the BEA-producing ability of our strain collection consisting of 100 *Fusarium* isolates from Hungary by studying the presence of beauvericin synthetase gene, and to investigate the origin of it. Another objective of this study was to determine the mycotoxin production ability of our strains focusing on the production of BEA, however, it was necessary to monitor the production of other occurring *Fusarium* toxins during the process in small bioreactor.

## Materials and Methods

### Molecular Biological Methods

The *Fusarium* strains used in this study are part of a collection established by the Hungarian University of Agriculture and Life Sciences, Institute of Aquaculture and Environmental Safety, Department of Environmental Safety between the years of 2011 and 2013. The isolates were mainly obtained from different parts of maize plants and were collected from different regions of Hungary with a total number of 100 *Fusarium* isolates (Supplementary Table S1).

The *DNA isolation* was carried out by a modified method generally applied in our Department of Environmental Safety. The cryopreserved strains were revitalized by inoculating them into a culture broth containing 1% peptone, 3% glucose, and 0.5% yeast extract. Approximately 4 cubic millimeters of fresh fungal mycelium were incubated in 3–3 ml of this liquid media for 72 h at room temperature, then 300 μL of 10% N-Lauryl-sarcosine was pipetted into them (anionic surfactant amphiphilic amino acid), which helps the cell lysis and to remove other proteins and cell wall fragments. Afterward, silica sand (274,739, white quartz, 50–70 mesh, Sigma-Aldrich, USA) was added to the tubes with a proportion of 2 g per sample and then the samples were scratched with a proculture plastic pestles (SP Bel-art®, Sigma-Aldrich, USA) micro-tube homogenizer system until forming a homogenous suspension. This mechanical action of the sand particles facilitated cell lysis by physically disrupting cell walls and membranes. After this process, the samples were incubated at 65 °C (Bio TDB-100, Dry Block thermostat—Biosan, Latvia) for 20 min, vortexed, and then placed on ice for 10 min. Then, 150 μL of sodium acetate (3 M, pH 5.5) was added to the samples to solubilize DNA, and the high salt concentration causes protein precipitation. Samples were centrifuged for 15 min at 9275×*g*, then 300–350 μL of the upper phase of each sample was transferred to sterile Eppendorf tubes and combined with 500 μL isopropanol (99.5%) which draws water away from the DNA, making it easier to precipitate. The addition of isopropanol is followed by re-centrifugation at 9275×*g* for 15 min, after which the liquid was poured out and 1 mL of ethanol (70%) was added and centrifuged for 5 min at 9275×*g*. This latter process was repeated again and each sample was dried, then the extracted DNA was dissolved in 30 μL of sterile MQ water. The DNA quality isolated from the samples was examined by 1% agarose gel electrophoresis.

*PCRs were carried out* for each reaction using a volume of 12.5 µL of Dream Taq PCR Master Mix (2X) (Thermo Fisher Scientific, USA), 8 µL of MQ water and 0.25 µM of forward primer and 0.25 µM of reverse primer, 1 mM of MgCl_2_, 1 μg of BSA. Later, ~ 10 ng of a DNA sample was added to the master mix.

A primer pair was chosen based on Stepien et al. [15] to amplify a domain homologous sequence to investigate the presence of the beauvericin synthetase gene (BEAS), which is responsible for the BEA production, Beas_1 as a forward (5′-TKGARCAGCGBCAYGAGACM) and Beas_2 as a reverse (5′-GGWCGRGGGAARTCRGTDGG). PCR conditions for BEAS gene were as follows: Denaturation start: 95 °C for 3 min; Annealing: 35 cycles of each for 30 s at 94 °C, 45 s at 50 °C to 61 °C, and 1 min 30 s at 72 °C; Extension: 10 min at 72 °C.

Another primer pair was chosen based on O’Donnel et al. [16] for species-level molecular identification of the isolates by investigating their translation-elongation factor-1alpha (tef1) gene ef1 as a forward (5′-ATGGGTAAGGARGACAAGAC-3′) and ef2 as a reverse (5′-GGARGTACCAGTSATCATG-3′). PCR conditions for TEF1 gene were as follows: Denaturation start: 95 °C for 3 min; Annealing: 35 cycles of each for 30 s at 95 °C, 30 s at 55 °C and 30 s at 72 °C; Extension: 3 min at 72 °C.

The Zymo research PCR clean up kit was used to remove unwanted by-products from the reaction. Those *Fusarium* spp. strains were chosen for the sequencing reaction and species-level identification, which showed positive BEAS PCR results and therefore had the BEA producing gene. The 23 *Fusarium* strains from the collection were: 1/1 F, 2/2 F, 4/2 F, 5/1 F, 6/1 F, 10/1 F, 12/2 F, 12/3 F, 13/3 F, 14/2 F, 22/1 F, 22/2 F, 23/3 F, 23/5 F, 24/3 F, 35/1 F, 37/2 F, 39/2 F, 41/1 F, 46/1 F, 53/1 F, 56/1 F, T698B. The sequencing PCR master mix for both BEAS and TEF1 genes has a final volume of 5 µL. It contained: 0.5 µL of Big Dye, 0.75 µL of Big Dye buffer (Thermo Fisher Scientific, USA), 0.25 µM of primer (BEAS), ~ 10 ng of template, and 2.5 µL of MQ water. The heat profile of the sequencing PCR was as follows: 28 times (96 °C for 10 min, 50 °C for 5 s, 60 °C for 4 min) and hold at 4 °C. The following step was for the recovery of fluorescently labeled DNA (Ethanol precipitation). An acetate mix was created for each sample: 3 µL of NaAC (3 M), 21 µL of MQ water, and 56 µL of ccEtOH (96%). The NaAC (Sodium acetate) (MW: 136.08 g/mol) is made from 80 ml of distilled water and 40.0824 g of CH_3_CO_2_Na x3H_2_O (MW: 136.08 g/mol). The pH was adjusted to 5.2 with glacial acetic acid. Then, the volume is adjusted to 100 mL with distilled water.

Later on, the acetate mix was added to the product of the sequencing PCR. The new mixtures were incubated for 10 min at room temperature. Samples were centrifuged for 25 min at 3910×*g*. After that, the supernatant was decanted and 180 µL of EtOH (70%) was added. Samples were centrifuged again for 20 min at 3910×*g* at a temperature of 4 °C. The residual supernatant was then completely decanted and the samples were centrifuged for one more minute, upside-down. Later, 20 µL of HI-DI formamide (Thermo Fisher Scientific, USA) was added. Finally, the samples were incubated for 24 h at 4 °C. Sanger sequencing of the amplicons was performed on a 3500 Genetic Analyzer (Applied bioscience) at BIOMI Ltd., Gödöllő. The sequencing data was corrected with the help of the application Finch TV version 1.4.0 (Geospiza, Inc.). The sequences were then assessed using the NCBI Blast, specifically Nucleotide blast (BLASTn), to search for identical nucleotide databases to our nucleotide sequences.

**Fig. 1:**
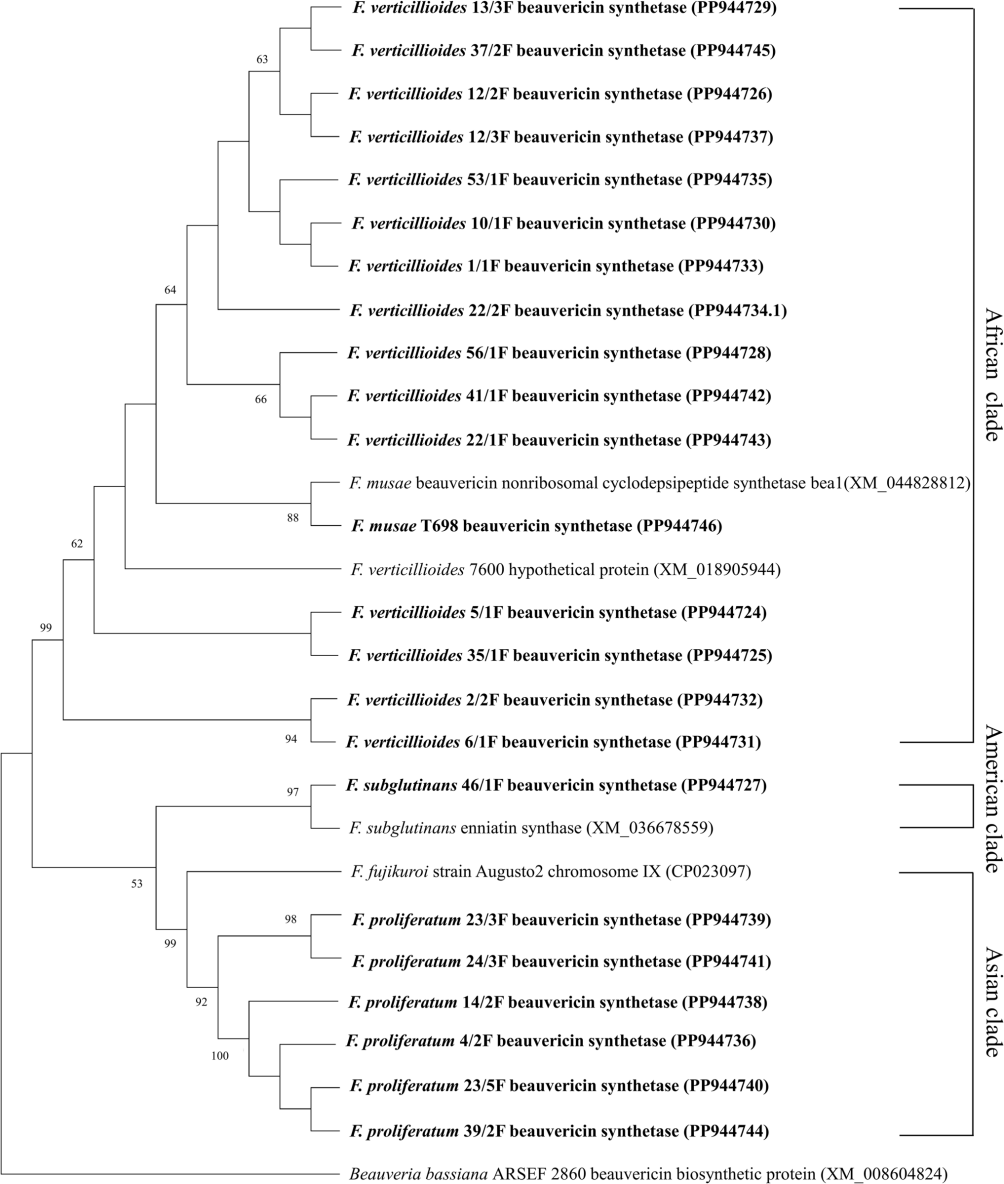
The maximum-likelihood phylogenetic tree done by Kimura method, according to the BEA synthetase gene (BEAS) of the 23 investigated strains compared with the closest similar BEAS sequence: XM_044828812 *F. musae*—African clade, XM_018905944 *F. verticillioides*—African clade, CP023097.1 *F. fujikuroi*—Asian clade, *XM_036678559 F. subglutinans*—American clade, XM_008604824.1 *Beauveria bassiana*. *Beauveria bassiana* ARSEF 2860 beauvericin biosynthetic protein (XM_008604824) was chosen as outgroup. Bootstrap values are given as percentages with 50% cut-off. Strains from the present study are in bold. NCBI accession numbers are in parenthesis

Phylogenetic analysis was performed using MEGA X software [17]. Within the MEGA X software ClustalW system was used for alignment. The phylogenetic tree was reconstructed maximum-likelihood [18] methods with Kimura’s two-parameter calculation model. Bootstrap analysis was used to evaluate tree topologies and distances by performing 1000 replicates (Fig. 1).

*Small-scale bioreactors* were used *to perform* the actual *toxin production* assays with strains characterized by BEA synthetase gene sequences. Glass containers containing 100 g of rice and 50 mL of distillated water (sterilized in an autoclave, 15 min at 120 °C) per sample were inoculated with a 1 mL spore suspension of approximately 1 million spores/mL, the determination of spore concentration was conducted by Bürker-chamber. The assembled experiment was incubated in darkness at 22.5 °C for 3 weeks, the matrixes were put into a drying chamber (48 h at 60 °C) and then homogenously ground with a grinder (BOSCH TSM6 A013B, Germany). Samples were quantitatively analyzed for the most typical *Fusarium* toxins like DON, FB1, FB2, T-2, HT-2 and BEA using a validated UHPLC-ESI-MS/MS analytical method. Measurements were conducted in three parallels.

### Preparation of Standard Solutions and Calibration

The stock solution of mycotoxin standard MIX (10.01 mg/kg DON; 1.00 mg/kg T2; 10.04 mg/kg HT-2; 3.03 mg/kg ZEA; 5.02 mg/kg FB1; 5.01 mg/kg FB2) was purchased from Romer Lab, Austria. The crystalline BEA standard was purchased from Fermentek Ltd, Israel, and was used for the preparation of the standard stock solution of BEA in a concentration of 10.0 mg/kg in MeOH. For quantitative analyses of the extracts of rice samples, an external calibration method was used by proportional dilution of the stock solutions (5–10–25–50–100 µL) by an acetonitrile/water/formic acid (75:25:0.1 V/V) solvent mixture.

### Sample Preparation

A total of 22 rice samples were extracted by a dilute-and-shoot method [19]. In the first step, 5.0 g of homogenously ground rice samples were weighed into 50 mL plastic tubes and supplemented with 25.0 mL of extraction solvent mixture [acetonitrile/water/formic acid (79:20:1 V/V%)]. Samples were extracted on a horizontal shaker (Certomat SII, Braun Hungary) for 60 min at a speed of 270 min^−1^, then centrifuged (Hermle Z 206 A, Dialab Ltd., Hungary) at 4000 g. Each sample’s 5 µL supernatant was removed and diluted to a total volume of 1000 µL with an acetonitrile/water/formic acid (75:24.9:0.1 V/V%) solvent mixture. After filtration through a 0.22 µm polytetrafluoroethylene filter, samples were injected in a UHPLC-ESI-MS/MS system [19]. All samples were analyzed in triplicates.

### Instrumentation and Method Validation

An Agilent 1290 HPLC system (Agilent Technologies) hyphenated with a triple-quadrupole mass spectrometer (Agilent Ultivo LC/TQ, Germany) with an electrospray ionization (ESI) interface was utilized. Agilent Mass Hunter Analyses software was used for instrument control, data acquisition, and processing. Chromatographic separation was performed using an Eclipse Plus C18 RRHD, 1.8 µm (2.1 × 50 mm) chromatographic column purchased from Agilent (Germany). For the chromatographic separation of mycotoxins, a binary mobile phase used comprised water (solvent A) and methanol (solvent B), both prepared with 5 mM ammonium-acetate and 0.1% acetic acid, according to Varga et al. [20].

The injection volume was 5 μL, the flow rate was 0.4 mL/min and the column temperature was set to 40 °C. The duration of chromatographic analyses was 7 min for one run using a gradient elution profile. The analysis was done in positive ion mode for DON, FB1, FB2, HT-2, T2, and BEA, while in negative mode in the case of ZEA with the following ion-source settings: gas temperature: 300 °C, gas flow: 9.0 L/min, nebulizer pressure: 40 psi and capillary voltage was 2500 and 4000 V for positive and negative mode, respectively. The mass spectrometer was operated in multiple reaction monitoring (MRM) mode measuring two transitions of each precursor (1 quantifier, 1 qualifier).

The analytical method was validated based on a concept by the IUPAC, Eurachem, SANTE and the European Union for single laboratory validation [21, 2002/657/EC, 22]. Limit of detection (LOD) values were 5.1, 12.0, 12.0, 0.7, 0.5, 1.6 and 0.05 µg/kg for DON, FB1, FB2, HT-2, T2, ZEA, and BEA, respectively. The limit of quantification (LOQ) values was 17.0, 39.0, 39.0, 2.4, 1.7, 5.2, and 0.2 µg/kg for the same analytes, respectively. The method is linear between the LOQ and 5000 µg/kg concentration range for all analytes. For recovery measurements, two spiking levels (namely at 2 and 5 times of LOQ) were investigated in 6 replicates. Extraction recovery was calculated by the same method as published by Varga et al. [19]. The recovery rates were between 85 and 120% for all analytes and the applicable values of RSD were below 10%.

### Statistical Analysis of Mycotoxin Production

All statistical analyses were performed with the Graphpad Prism 8.0.1 software package. For normality analysis Shapiro–Wilk test was performed, then analysis of variance (ANOVA) was used to compare the mycotoxin concentrations found in the culture extracts of the strains. Individual toxin concentrations of the extracts and the relationship between the concentration of the identified compound and the clades were assessed by Tukey’s multiple comparisons test. Isolates were assumed to be random samples.

## Results

Out of 100 *Fusarium* strains, 23 isolates showed a positive PCR result with the BEAS primers. Based on TEF1α sequence homology, these strains could be classified into 4 species: *F. verticillioides*, *F. proliferatum*, *F. musae* and *F. subglutinans*. According to O’Donnell et al. [23] *Fusarium fujikuroi* species complex (FFSC) can be classified into three biogeographic clades, i.e. the African, the American and the Asian clade, based upon the origins. It can be concluded that 70% of the investigated strains originate from African clades (16/23, *F. verticillioides* and *F. musae*). The second largest clade is the Asian clade since 26% (6/23, *F. proliferatum*) of the strains belong to this monophyletic group. Finally, 4% (1/23, *F. subglutinans*) of these Fusarium strains are a part of the American clade according to the similarity of the TEF1α identifications (Table 1).

**Table 1:** The identification results of the investigated strains via TEF1α region showing the Geographical origin of the strains according to the study of O’Donnell et al. [23], compared by the BEAS gene sequence comparison results

Strain ID	Identification via TEF1α region	The TEF1α sequences were deposited in GenBank under these accession numbers	Geographical origin of the FFSC species complex according the TEF1α identification	The closest species and its geographical origin according to TEF1α region	The closest species and its geographical origin according the BEAS gene sequence comparison	The BEAS gene sequences were deposited in GenBank under these accession numbers
46/1 F	*F. subglutinans*	PQ468708	American clade	ON557397.1 *F. subglutinans*—American	XM_036678559 *F. subglutinans—American*	PP944727.1
4/2 F	*F. proliferatum*	PQ468717	Asian clade	MN784807.1 *F. proliferatum*—Asian	CP023097.1 *F. fujikuroi—Asian*	PP944736.1
14/2 F	*F. proliferatum*	PQ468719	Asian clade	MN861776.1 *F. proliferatum*—Asian	CP023097.1 *F. fujikuroi—Asian*	PP944738.1
23/3 F	*F. proliferatum*	PQ468720	Asian clade	MH496039.1 *F. proliferatum*—Asian	CP023097.1 *F. fujikuroi—Asian*	PP944739.1
23/5 F	*F. proliferatum*	PQ468721	Asian clade	KX215079.1 *F. proliferatum*—Asian	CP023097.1 *F. fujikuroi—Asian*	PP944740.1
24/3 F	*F. proliferatum*	PQ468722	Asian clade	MH179131.1 *F. proliferatum*—Asian	CP023097.1 *F. fujikuroi—Asian*	PP944741.1
39/2 F	*F. proliferatum*	PQ468725	Asian clade	MN784802.1 *F. proliferatum*—Asian	CP023097.1 *F. fujikuroi—Asian*	PP944744.1
1/1 F	*F. verticillioides*	PQ468714	African clade	OL828714.1 *F. verticillioides*—African	XM_018905944 *F. verticillioides—African*	PP944733.1
2/2 F	*F. verticillioides*	PQ468713	African clade	MN861780.1 *F. verticillioides*—African	XM_018905944 *F. verticillioides—African*	PP944732.1
5/1 F	*F. verticillioides*	PQ468705	African clade	ON692980.1 *F. verticillioides*—African	XM_018905944 *F. verticillioides—African*	PP944724.1
6/1 F	*F. verticillioides*	PQ468712	African clade	MN861780.1 *F. verticillioides*—African	XM_018905944 *F. verticillioides—African*	PP944731.1
10/1 F	*F. verticillioides*	PQ468711	African clade	MT010990.1 *F. verticillioides*—African	XM_018905944 *F. verticillioides—African*	PP944730.1
12/2 F	*F. verticillioides*	PQ468707	African clade	OL828704.1 *F. verticillioides*—African	XM_018905944 *F. verticillioides—African*	PP944726.1
13/3 F	*F. verticillioides*	PQ468710	African clade	PP196432.1 *F. verticillioides*—African	XM_018905944 *F. verticillioides—African*	PP944729.1
22/1 F	*F. verticillioides*	PQ468724	African clade	MZ404064.1 *F. verticillioides*—African	XM_018905944 *F. verticillioides—African*	PP944743.1
35/1 F	*F. verticillioides*	PQ468706	African clade	OL828714.1 *F. verticillioides*—African	XM_018905944 *F. verticillioides —African*	PP944725.1
53/1 F	*F. verticillioides*	PQ468716	African clade	OL828714.1 *F. verticillioides*—African	XM_018905944 *F. verticillioides—African*	PP944735.1
56/1 F	*F. verticillioides*	PQ468709	African clade	PP196432.1 *F. verticillioides*—African	XM_018905944 *F. verticillioides—African*	PP944728.1
12/3 F	*F. verticillioides*	PQ468718	African clade	PP196445.1 *F. verticillioides*—African	XM_018905944 *F. verticillioides—African*	PP944737.1
37/2 F	*F. verticillioides*	PQ468726	African clade	MT707623.1 *F. verticillioides*—African	XM_018905944 *F. verticillioides—African*	PP944745.1
22/2 F	*F. verticillioides*	PQ468715	African clade	PP196438.1 *F. verticillioides*—African	XM_018905944 *F. verticillioides—African*	PP944734.1
41/1 F	*F. verticillioides*	PQ468723	African clade	PP196447.1 *F. verticillioides*—African	XM_018905944 *F. verticillioides—African*	PP944742.1
T698B	*F. musae*	OL365732	African clade	OL365732.1 *F. musae*—African	XM_044828812 *F. musae*—*African*	PP944746.1

The study also revealed whether the phylogeny reconstruction of the BEAS sequences of our *Fusarium* strains followed the biogeographical origins of *Fusarium* species defined by O’Donnell et al. This kind of segregation may provide insights into a broader evolutionary relationship within the species complex [24]. The comparison of the BEA synthetase gene sequences in the NCBI international database allowed us to infer the extent of the BEA-producing species predominant in the Carpathian Basin, especially Hungary.

Comparison of the BEAS gene of the isolated strains with the maximum-likelihood phylogenetic tree using the Kimura method yielded similar results as the TEF1α clustering. However, we could only distinguish two main groups: the beauvericin synthetase genes belonging to the strains *F. verticillioides* and *F. musae* formed one large cluster, while *F. proliferatum*, *F. fujikuroi* and *F. subglutinans* BEAS gene formed the other cluster, although within this branch the Asian and American origins were clearly distinguishable (Fig. 1).

The toxin production capacity of strains carrying the BEAS gene was determined by small-scale bioreactor experiments. The toxin production of Fusarium T698B strains could not be confirmed due to revitalization problems. The presence of BEA, FB1 and FB2 was detectable in all samples, while DON, T2, HT-2 and ZEA toxins were not found in any case, the measured mycotoxin concentrations can be seen in Table 2.

**Table 2:** The BEA, FB1, FB2, DON, T2, HT-2 and ZEA producing ability of the strains according to the UHPLC-ESI-MS/MS measurement, the results were analyzed by Tukey’s multiple comparisons test within clades, results with * are significant (*P* value < 0.0001)

Strain ID	Identification via TEF1α region	BEA (mg/kg)	FB1 (mg/kg)	FB2 (mg/kg)	FB1/FB2 ratio	DON (mg/kg)	T2 (mg/kg)	HT-2 (mg/kg)	ZEA (mg/kg)
46/1 F^A^	*F. subglutinans*	6.9 ± 1.0*	4.9 ± 2.1* < 0.0001	0.6 ± 0.1*	5.0	N.D.	N.D.	N.D.	N.D.
4/2 F^B^	*F. proliferatum*	1370 ± 117.8*	331 ± 34.2**	53 ± 7.0**	6.3	N.D.	N.D.	N.D.	N.D.
14/2 F^C^	*F. proliferatum*	731 ± 135.4*	2507 ± 116.0*	322 ± 34.2*	7.8	N.D.	N.D.	N.D.	N.D.
23/3 F^D^	*F. proliferatum*	1827 ± 58.7*	330 ± 23.3**	76 ± 3.2**	4.3	N.D.	N.D.	N.D.	N.D.
23/5 F^E^	*F. proliferatum*	452 ± 54.1**	601 ± 72.3*	138 ± 2.7*	4.4	N.D.	N.D.	N.D.	N.D.
24/3 F^F^	*F. proliferatum*	3131 ± 153.0*	372 ± 19.4**	93 ± 7.6*	4.0	N.D.	N.D.	N.D.	N.D.
39/2 F^G^	*F. proliferatum*	587 ± 60.5**	352 ± 27.5**	44 ± 10.1**	8.0	N.D.	N.D.	N.D.	N.D.
1/1 F^H^	*F. verticillioides*	1.1 ± 0.2	3640 ± 453.7**	1201 ± 98.0**	3.0	N.D.	N.D.	N.D.	N.D.
2/2 F^I^	*F. verticillioides*	8.2 ± 0.4**	306 ± 29.0	92 ± 2.8	3.3	N.D.	N.D.	N.D.	N.D.
5/1 F^J^	*F. verticillioides*	13 ± 1.5*	50 ± 3.7	7.9 ± 2.4	4.5	N.D.	N.D.	N.D.	N.D.
6/1 F^K^	*F. verticillioides*	0.5 ± 0.0	3287 ± 15.7**	1052 ± 45.7**	3.1	N.D.	N.D.	N.D.	N.D.
10/1 F^L^	*F. verticillioides*	1.0 ± 0.2	238 ± 20.0	78 ± 5.1	3.1	N.D.	N.D.	N.D.	N.D.
12/2 F^M^	*F. verticillioides*	2.4 ± 0.2	160 ± 7.3	72 ± 9.5	2.2	N.D.	N.D.	N.D.	N.D.
13/3 F^N^	*F. verticillioides*	0.6 ± 0.1	601 ± 148.5	179 ± 66.9	3.4	N.D.	N.D.	N.D.	N.D.
22/1 F^O^	*F. verticillioides*	1.3 ± 0.2	552 ± 28.7	193 ± 34.2	2.9	N.D.	N.D.	N.D.	N.D.
35/1 F^P^	*F. verticillioides*	61 ± 1.1*	41 ± 5.0	11 ± 2.6	5.1	N.D.	N.D.	N.D.	N.D.
53/1 F^Q^	*F. verticillioides*	2.5 ± 0.1	4393 ± 678.2*	1390 ± 153.4*	3.2	N.D.	N.D.	N.D.	N.D.
56/1 F^R^	*F. verticillioides*	10 ± 0.4**	83 ± 4.4	27 ± 3.0	4	N.D.	N.D.	N.D.	N.D.
12/3 F^S^	*F. verticillioides*	2.4 ± 0.3	392 ± 66.7	104 ± 11.0	3.8	N.D.	N.D.	N.D.	N.D.
37/2 F^T^	*F. verticillioides*	0.5 ± 0.0	208 ± 17.9	79 ± 10.8	2.6	N.D.	N.D.	N.D.	N.D.
22/2 F^U^	*F. verticillioides*	20 ± 2.0*	422 ± 64.7	139 ± 25.2	3.0	N.D.	N.D.	N.D.	N.D.
41/1 F^V^	*F. verticillioides*	0.6 ± 0.2	147 ± 6.7	41 ± 9.9	3.6	N.D.	N.D.	N.D.	N.D.
T698B^W^	*F. musae*	N.D.	N.D.	N.D.	N.D.	N.D.	N.D.	N.D.	N.D.

Results marked with ** are not significantly different from each other, but significantly different from the other results within the same clade (*P* value < 0.0001)

^*^Significant with all the other results (*P* value < 0.0001); ** are not significantly different from each other, but significantly different from the other results within the same clade (*P* value < 0.0001). Additional significant pairwise differences: *F. verticillioides*: H–Q (*P* = 0.012, FB1), H–Q (0.0094, FB2), J–O (*P* = 0.0145, FB2), J–N (*P* = 0.0318, FB2), O-P (*P* = 0.0119, FB2), O–R (*P* = 0.0253, FB2), P-N (*P* = 0.0262, FB2), *F. proliferatum* & *F. subglutinans*: B–E (*P* = 0.0005, FB1), D–E (*P* = 0.0005, FB1), E–F (*P* = 0.0026, FB1), E–G (*P* = 0.0012, FB1), B–F (*P* = 0.0462, FB2), B–A (*P* = 0.0067, FB2), D–E (*P* = 0.0016, FB2), D–A (*P* = 0.0002, FB2), E–F (*P* = 0.0209, FB2), F–G (*P* = 0.0116, FB2), G–A (*P* = 0.0266, FB2), B–D (*P* = 0.0007, BEA), C–E (*P* = 0.0403, BEA), E–A (*P* = 0.001, BEA)

According to the LOQ, BEA production ability can be reported for all of the 22 strains that were tested in bioreactor, with several able to produce exceptionally high values of BEA. There was significant difference among the Asian and African (*P* < 0.0001); Asian and American clades (*P* = 0.0002) BEA production level. The highest BEA levels were observed in the *F. proliferatum* strains (belonging to the—Asian clade), with a range of 452–3131 μg/kg. BEA production was also observed in *F. verticillioides* strains, but the production level was not outstanding. Members of the African clade were capable of producing BEA under 20 mg/kg except one strain, which was produced 61 mg/kg. However, these strains tended to produce higher levels of fumonisin B1 and B2. For FB1, 18 strains out of 22 were able to produce concentrations higher than 100 μg/kg, while in the case of FB2, the measured values were above this concentration in 9 cases. The highest amount of FB1 (4393 mg/kg) and FB2 (1390 mg/kg) was detected in *F. verticillioides* strain 53/1 F—belonging to the African clade. Among the clades there was no significant difference in the level of FB1 and FB2 production.

## Discussion

The objective of this study was to investigate the beauvericin and related mycotoxin-producing capabilities of a *Fusarium* strain collection, consisting of 100 *Fusarium* species isolated from all geographical regions of Hungary. The member of the collection was mainly isolated from maize plants (Supplementary Table S1). The study focused on the BEA production ability, which was evaluated by two steps: first by a PCR method, detecting the BEA synthetase gene by a specific primer. Among the 100 *Fusarium* strains, 23 have the gene responsible for the production of BEA which could also indicate a relatively high prevalence of BEA in Hungary. A study conducted by Santini et al. [25] revealed that the contamination with BEA in terms of concentration level is higher in Southern Europe compared with Central or Northern Europe, which can be explained by the higher prevalence of BEA-producing fungi in Southern Europe. According to this review, the highest reported concentration of BEA (59 mg/kg) was detected in naturally contaminated maize from Morocco, which as a Mediterranean country contradicts Reyes-Velázquez’s claim that colder temperature regions would be more favorable for BEA production [25, 26].

BEA has been labeled as an emerging mycotoxin because of its important incidence in a wide range of food crops like wheat, barley, oats, rye, and rice, also numerous studies have demonstrated that BEA-producing *Fusarium* species can be isolated from crops of significant economic importance (rice, maize, wheat) and in vitro BEA-producing ability of these isolates has been frequently observed [26–28]. In fact, BEA has been detected in a multitude of countries lately, such as China, Norway, South Africa, Morocco, Spain, Italy, and Croatia [29]. Serrano et al. [30] performed a study in which 265 samples formed from cereal grains and cereal-based products were collected from different Mediterranean countries (Morocco, Italy, Spain, and Tunisia). Furthermore, the occurrence and concentration of BEA and 13 other mycotoxins in those cereal products were studied and the results showed that BEA was one of the most predominant mycotoxin with a presence in 27% of the positive samples at a concentration range of 2.1–844 µg/kg [30]. Besides, Streit et al. [31] detected 139 fungal mycotoxins from 83 feed samples collected primarily in Europe (71 samples): Hungary (19 samples), Austria (17 samples), Denmark (15 samples), while the rest of the samples were obtained from Ukraine, Belgium, Italy, Russia, and the UK. The results of the study showed that BEA was found in 98% of the samples and was the most common mycotoxin. In the same study, six maize cob samples showing different amounts of mold infection were collected from the same field in southern Austria during 2010 and 2012. The concentrations of different mycotoxins were studied and the results showed that all six maize cobs contained BEA with quite alarming concentrations ranging from 57 to 1591 µg/kg [31]. As for feed, BEA was detected in corn-dried distiller grains in Thailand [32], as well as in farm silages in Ireland [33]. Between 2006 and 2007, 27% of feed ingredients in Korea were contaminated with BEA [34].

Wheat is mainly infected by *F. poae*, which is associated with *Fusarium* head blight. According to Somma et al. [28], study on *Fusarium poae* revealed that 95% (77/81) of the tested *F. poae* isolates were found to be capable of producing BEA within a range from 2 to 2655 µg/g in Northern Italy. Stanciu et al. [35] in 2017 reported a study from Romania, where 133 wheat samples were investigated and BEA was detected only in 3 samples in a concentration between 0.07 and 9.1 µg/kg. In a recent study, Xu et al. [36] analyzed 769 samples to investigate the natural occurrence of BEA in wheat, 41 (5.33%) of which detected beauvericin with a range of 0.09–387.67 µg/kg concentration. Overall, there is evidence to suggest that *Fusarium* species that are associated with *Fusarium* head blight of wheat can have the potential ability to produce BEA, however, the existing literature suggests that it is not of a major importance.

Among our strains derived from maize samples, the *F. verticilloides* and *F. proliferatum* species were the most dominant based on TEF1α sequence identification. Numerous studies have documented *F. verticilloides* and *F. proliferatum* (both belong to the FFSC species complex) as the most prevalent fungi associated with maize, causing the *Fusarium* kernel rot of the plant [37]. Reyes-Velázquez et al. conducted a study on the natural FBs, BEA contamination of different maize hybrids in Jalisco (Mexico), and their findings revealed the presence of *Fusarium* species, with *F*. *verticillioides*, *F*. *proliferatum* and *F*. *subglutinans* being the most prevalent natural isolates. This study was the first to demonstrate co-occurrence of BEA (with a range of 300–400 ng/g mean value per maize hybrids) and FBs (17–606 ng/g) in Mexico. These results showed that *F. verticilloides isolates were not able to produce BEA*, but only FBs, whereas *F*. *proliferatum* and *F*. *subglutinans* isolates produced BEA. All of this suggests, that BEA has been detected mainly in maize, on this crop plant the *Fusarium* species belonging to the *FFSC* has been identified as the predominant contaminant [24, 26, 36].

Nicolli et al. [27] conducted a study on the diversity and toxin-producing ability of 100 isolates from the FFSC species complex isolated from Brazilian *rice*. From the tested isolates only the *F. verticillioides strains could not produce BEA*. The majority of isolates of *F. fujikuroi*, *F. proliferatum* and *F. verticilloides* were found to produce FBs. The presence of members of the FFSC species complex is common on rice, especially *F. proliferatum, F. verticilloides* and *F. fujikuroi*. This study further demonstrates the co-occurrence of FBS and BEA not only in maize-based crops but also in rice.

All of this suggests, that BEA has been detected mainly in maize, on this crop plant the *Fusarium* species belonging to the FFSC has been identified as the predominant contaminant [24, 26, 36]. On wheat *F. poae* has been identified as a frequent pathogen agent, according to the literature with a much lower BEA-producing occurrence [35, 36].

The second step of the BEA producing evaluation was to set up small-scale bioreactors for mycotoxin production and the samples were measured by UHPLC-ESI-MS/MS. Relevant publications on laboratory-scale BEA production were reviewed, and in most cases, toxin production occurred at pH 7 and a temperature optimum between 25 and 27 °C, as the BEAS enzyme was inactive above 30 °C [38]. According to Kostecki et al. [39], the highest amounts of BEA were produced on rice and wheat (among barley, wheat, maize, rice, rye, oat substrates). In summary, there seems to be no significant difference between the in vitro BEA production studies documented in the literature (Supplementary Table S2) and the methods and parameters of the present study, except that our study was performed at a slightly lower temperature of 22.5 °C. However, the toxin concentrations experienced demonstrate that this temperature is appropriate for the intended studies.

In our experiment, we decided to follow the method of Urbaniak et al. from 2020 [5], where they used rice as a matrix for BEA production. Urbaniak et al. tested 6 *Fusarium* strains, and 2 were not producing BEA at all. Their study shows that the highest producers of BEA were *F. proliferatum* and *F. nygamai*, with 90 and 22 µg/g concentration, respectively.

In our case we proceeded the test with 22 strains where the BEAS gene was present, and all the investigated strains produced BEA on rice matrix above the UHPLC-ESI-MS/MS detection limit (0.05 µg/g). The *F. proliferatum* (7) were the best BEA producer, the highest concentration was 3131 mg/kg*.* The *F. verticillioides* strains (14) were also producing BEA between 0.5 and 61 mg/kg. Compared to the present study, the studies of Moretti et al. [40] differ in the toxin production substrate (they used maize instead of rice), the incubation temperature (25 °C instead of 22.5 °C) and the strains studied (only *F. subglutinans*). 60% of the *F. subglutinans* strains they studied were capable of BEA production in amounts ranging from 5 to 200 mg/kg. Logrieco et al. [41] also investigated the BEA producing capabilities of 94 *Fusarium* strains belonging to 25 taxa of the *Fusarium* genus. Their results showed that BEA is one of the most widely produced mycotoxins by *Fusarium* species, 33 of the 94 strains produced BEA (35%). In addition, the highest BEA concentration (3200 mg/kg) was detected in a *F. oxysporum* strain isolated from maize stalks in Poland. Moreover, other high concentrations of BEA were found in *Fusarium* strains coming from Germany, England, Iran, Philippines, Panama and Ecuador with different original plant hosts like potato, grape, maize, string beans and musa fruit [41].

From the bioreactor experiments, the most frequent *Fusarium* toxins were investigated such as DON, T2, HT-2, ZEA, FB1, and FB2. Except for BEA, FB1, and FB2, no other toxins were observed above the detection limit.

According to the TEF1α sequence identification the strains can be grouped into African (*F. verticillioides, F. musae*), Asian (*F. proliferatum*) and American (*F. subglutinans*) clades of origin [23]. The biggest group was the African clade (16 strains, 15 were *F. verticillioides* and 1 was *F. musae*). The members of the African clade were producing BEA in a smaller amount 0.5–61 mg/kg, although these strains produced the highest FBs concentrations: 4393 mg/kg FB1 and 1390 mg/kg FB2. The Asian clade was the second biggest geographic group with 7 strains, all of them were *F. proliferatum* species, with the highest BEA-producing abilities reaching 3131 mg/kg (all strains showed BEA concentration over 400 mg/kg).

The direct environmental factors that influence BEA production are not fully understood currently, but the difference in the production of the compound may require a genetic approach, either in terms of genetic polymorphism in the genes involved in the biosynthesis or differences in the regulatory elements. According to Niehaus et al. (2016) synthesis of BEA is influenced not only by BEAS but also by a gene encoding a ketoisovalerate-reductase (beas2—which has a role in precursor creating), an ABC transporter (beas3) and a Zn(II)2 Cys6 transcription factor (beas4) [42]. The synthesis of BEA is dependent on all of these genes probably, however, the rate of production of the compound relies on the expression levels of these genes, which could be verified by transcriptomic methods. The different BEA production levels among the investigated *F. proliferatum* strains can be explained by this gene expression heterogeneity.

Interestingly, several samples had high concentrations of BEA concurrent with lower concentrations of FBs mycotoxin production. For example, the 24/3 F strain (belonging to the Asian clade of the FFSC) produced 3131 mg/kg of BEA and synthesized 371 mg/kg of FB1, 10% of the BEA concentration, and even less FB2 (92 mg/kg). Presumably, different gene clusters are involved in the biosynthesis of different toxins and only one type of secondary metabolite could be produced at the same time in a higher amount. All investigated strains were able to produce FB1 in varying concentrations, between 4.9 and 4393 mg/kg. The highest obtained FB1 value was 4393 mg/kg synthesized by strain 53/1 F, belonging to the African clade of the FFSC. Regarding FB2, less was detected, ranging from 0.6 to 1390 mg/kg. According to our results, the production of FB1 follows a nearly parallel correspondence with FB2 toxins, with a median ratio value of 3.3. This indicates that in the majority of the cases, nearly three times as much FB1 was produced than FB2. Mycotoxin production is frequently accompanied by the assembly of co-expressing genes embedded in a complete gene cluster. However, Proctor et al. [43] demonstrated in their study that the 16-gene fumonisin biosynthetic gene cluster can be horizontally transferred between *Fusarium* species. As previously mentioned, *F. verticilloides* (African clade of FFSC and *F. proliferatum* (Asian clade of FFSC) might be the most common fungi associated with maize infection, as major causative agents of *Fusarium* kernel rot in maize, and according to Marín et al. [37] the occurrence of the species is positively correlated with FB contamination in maize, especially in warm, dry years. Although these species could germinate in a wide range of temperatures 5–37 °C in corn extract agar, their optimal temperature to produce FBs can differ. In the case of *F. verticilloides* it is 20 to 30 °C, while in the case of *F. proliferatum* it is at 15 °C according to Marín et al. [37]. According to the results of the 2023 World Mycotoxin Survey [11], very high levels of FBs contamination were detected in the Sub-Saharan region (68%), Middle East and North-African region (94%), in South Africa (54%), in South-East Asia (94%), in Central America (78%), and in South-East Asia (94%), therefore it can be hypothesized that *F. verticillioides* strains with a higher temperature optimum for FB biosynthesis and belonging to the African clade are likely to be more prevalent in these countries. Our bioreactor experiment results are proof of BEA, FB1 and FB2 co-occurrence in the case of *Fusarium* strains living on crop plants. In the case of FB1 and FB2 there is a high human health risk, therefore almost every grain stock is evaluated for FBs. For BEA there is no limit value estimated by the EFSA, because it is not considered to be harmful to humans and animals at the moment. Because of that, presence of BEA is not frequently measured in grain and other food materials. This can provide a hidden increased risk, because one of the abilities of the BEA is the potentiating effect, where BEA is increases the cellular uptake of the co-occurring chemicals in the different matrixes. This could happen because, BEA can increase ion permeability in biological membranes by forming a complex with essential cations (Ca^2+^, Na^+^, K^+^), which may affect ionic homeostasis [44, 45], this can be an explanation for the synergic cytotoxic effects were demonstrated on PK15 cells exposed to BEA and ochratoxin A (OTA), FB1 and OTA, BEA and FB1 and OTA [46].

During food industrial processes, the contamination of FB1 and FB2 is decreased by 60% due to the heat treatment by extrusion [47]. The chemical structure of BEA is degrading over 95 °C, according to the heat treatment, BEA contamination is also decreasing [8].

It can be concluded that even lower mycotoxin contamination may pose an increased risk to human and animal consumption of feed and raw food products, due to the possible potentiating effect of BEA. This phenomenon should be investigated in the future studies for better food and feed safety.

## Conclusions

Hungary’s agriculture is focusing on the production of corn and wheat, which are the most dominant crops with their 1–1 million hectare sowing area, with an average annual production of 6 million tonnes of corn and 5 million tonnes of wheat. The quality of that grain crop is deeply affecting the profitability of the agricultural sector. In Hungary, the temperature conditions are in a transformation during the vegetation period of corn, it is a mixture of the southern hot and northern cold circumstances, with a very high temperature fluctuation of warm and cold weeks till the early summer (end of June) because of the climate change recently. This means that Hungary’s and surrounding regions (Carpathian Basin) maize production is highly endanger by the FBs producing *Fusarium* strains belonging to the African clade of the FFSC, which strains are able to co-produce BEA at the same time.

In the Carpathian Basin, we found a 23% BEA gene abundance among 100 *Fusarium* strains mainly causing maize infections. These 23 *Fusarium* strains have been classified into 4 different species (*F. verticillioides*, *F. proliferatum, F. musae* and *F. subglutinans*) which can be classified into 3 different monophyletic groups, an African, an American, and an Asian clade according to the TEF1α sequence identification. The phylogeny reconstruction of the BEAS sequences of our *Fusarium* strains mostly followed the biogeographical origins of *Fusarium* species. However, we could only distinguish two main groups: the beauvericin synthetase genes belonging to the African clade strains (*F. verticillioides* and *F. musae*) formed one cluster, while Asian and American clade member (*F proliferatum*, and *F. subglutinans*) BEAS gene formed the other cluster, although within this branch the Asian and American origins were clearly distinguishable.

In our study *F. proliferatum* strains belonging to the Asian clade have shown the highest BEA production ability (up to 3131 mg/kg), while the most abundant species were the *F. verticillioides* (African clade), with an order of magnitude less BEA production (up to 61 mg/kg). In all BEA-producing strain we could measure FB production at the same time, however, the further tested DON, T2, HT-2, ZEA toxins were not detectable. A *F. verticillioides* strain produced the highest concentration of FBs (4393 mg/kg FB1 and 1390 mg/kg FB2). This co-occurrence of the BEA and FB was not investigated in Hungary previously, although, this coincidence is might holding a hidden food safety risk. The European Union has set a maximum tolerable daily intake of FBs. Meanwhile, BEA does not have a food safety limit currently, but some studies suggest that it can increase the toxicity of other bioactive substances, and therefore potentially also the co-produced FB mycotoxins. Consequently, the food safety risk associated with these mycotoxins may be subject of further reconsideration. Future studies should investigate the synergic effect of BEA on co-occurring mycotoxins.

## Supplementary Information

Below is the link to the electronic supplementary material.

Supplementary file1 (TIF 158 KB)

Supplementary file2 (PNG 50 KB)

Supplementary file3 (DOCX 25 KB)

Supplementary file4 (DOCX 22 KB)

## Abbreviations

BEA: Beauvericin
BEAS: Beauvericin synthetase
ZEA: Zearalenon
FB1: Fumonisin B1
FB2: Fumonisin B2
DON: Deoxynivalenol
T2: T2 Toxin
HT-2: HT-2 Toxin
FFSC: *Fusarium fujikuroi* species complex
UHPLC-ESI-MS/MS: Ultra-high performance liquid chromatography-electrospray ionization tandem mass spectrometry
