# Supplementary figures and images for: Co-Occurrence of Beauvericin and Fumonisin Producing Ability of Fusarium Strains Isolated from Crop Plants in Hungary
Source: Curr Microbiol. 2025 May 23;82(7):302. doi: 10.1007/s00284-025-04243-9 (PMC12101999; doi:10.1007/s00284-025-04243-9)

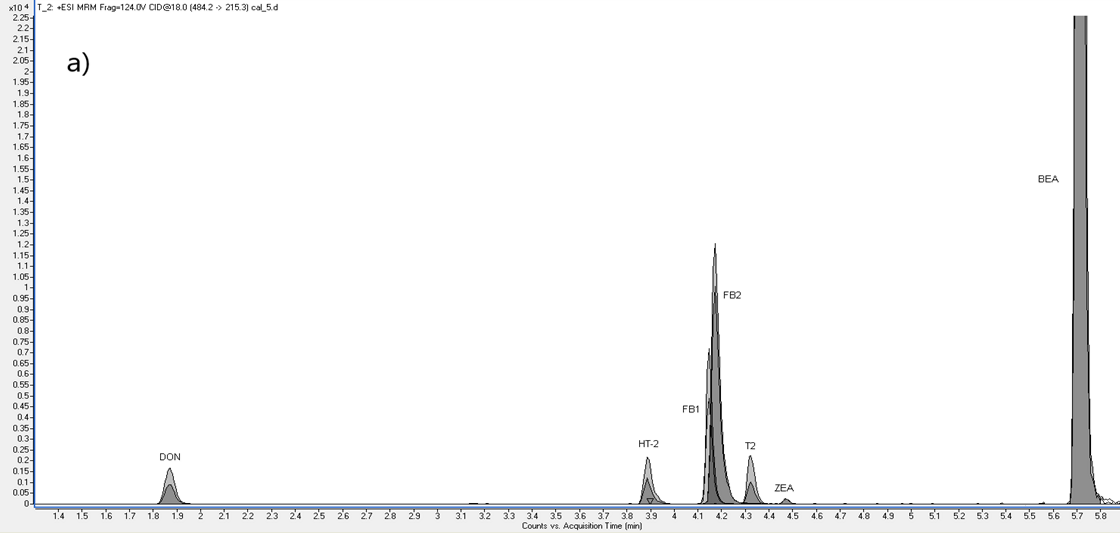

Supplement: Supplementary file 1 — Supplementary file1 (TIF 158 KB) [file 284_2025_4243_MOESM1_ESM.tif]

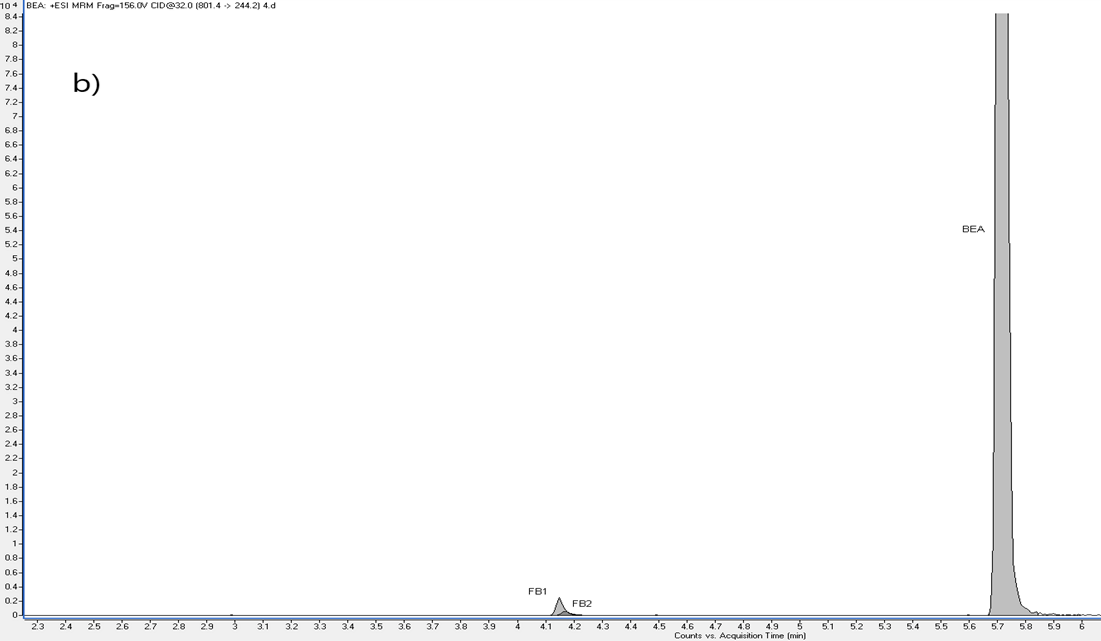

Supplement: Supplementary file 2 — Supplementary file2 (PNG 50 KB) [file 284_2025_4243_MOESM2_ESM.png]
