# Supplementary material for: Co-Occurrence of Beauvericin and Fumonisin Producing Ability of Fusarium Strains Isolated from Crop Plants in Hungary
Source: Curr Microbiol. 2025 May 23;82(7):302. doi: 10.1007/s00284-025-04243-9 (PMC12101999; doi:10.1007/s00284-025-04243-9)
Supplement: Supplementary file 3 — Supplementary file3 (DOCX 25 KB) [file 284_2025_4243_MOESM3_ESM.docx]

Article title: Co-occurrence of beauvericin and fumonisin producing ability of Fusarium strains isolated from crop plants in Hungary

Journal name: Current Microbiology

Authors: Ákos Suhajda, Mohammed Al-Nussairawi, Ines Amara, Csilla Sörös, Rita Tömösközi-Farkas, Balázs Kriszt, Milán Farkas, Mátyás Cserháti

Corresponding author: Balázs Kriszt, [Kriszt.Balázs@uni-mate.hu](mailto:Kriszt.Balázs@uni-mate.hu)

| **Specimen ID** | **City of isolation** | **County** | **Host** | **Identification** | **NCBI accession number** |
| --- | --- | --- | --- | --- | --- |
| 2885 | Debrecen | Hajdú-Bihar | Maize (Hungary) | *Fusarium* sp. (morphological) |  |
| 2886 | Kaposfüred | Somogy | Maize (Hungary) | *Fusarium* sp. (morphological) |  |
| 2887 | Kaba | Hajdú-Bihar | Maize (Hungary) | *Fusarium* sp. (morphological) |  |
| 2888 | Bicsérd | Baranya | Maize (Hungary) | *Fusarium* sp. (morphological) |  |
| **5/1F** | **Dánszentmiklós** | **Pest** | **Maize (Hungary)** | ***F. verticillioides* (morphological and PCR based-Tef1α)** | **PQ468705** |
| 946 | Budapest | Pest | Maize (Hungary) | *Fusarium* sp. (morphological) |  |
| T700B | Budapest | Pest | Pitaya (Vietnam) | *Fusarium* sp. (morphological) |  |
| T690D | Budapest | Pest | Ananas (Costa Rica) | *Fusarium* sp. (morphological) |  |
| **T698B** | **Budapest** | **Pest** | **Banana (Dominican Republic)** | ***F. musae* (morphological and PCR based-Tef1α)** | **OL365732** |
| 3187 | Vácszentlászló | Pest | Maize (Hungary) | *Fusarium* sp. (morphological) |  |
| **3233** | Vácszentlászló | Pest | Maize (Hungary) | *Fusarium* sp. (morphological) |  |
| 3254T | Vácszentlászló | Pest | Maize (Hungary) | *Fusarium* sp. (morphological) |  |
| 38/1F | Makád | Pest | Maize (Hungary) | *Fusarium* sp. (morphological) |  |
| 15/1F | Pest | Pest | Maize (Hungary) | *Fusarium* sp. (morphological) |  |
| 26/1F | Pilis | Pest | Maize (Hungary) | *Fusarium* sp. (morphological) |  |
| **35/1F** | **Makó** | **Csongrád-Csanád** | **Maize (Hungary)** | ***F. verticillioides* (morphological and PCR based-Tef1α)** | **PQ468706** |
| 1/2F | Mezőkeresztes | Borsod-Abaúj-Zemplén | Maize (Hungary) | *Fusarium* sp. (morphological) |  |
| 8/1F | Pest county | Pest | Maize (Hungary) | *Fusarium* sp. (morphological) |  |
| 36/2F | Pócsmegyer | Pest | Maize (Hungary) | *Fusarium* sp. (morphological) |  |
| **12/2F** | **Pest county** | **Pest** | **Maize (Hungary)** | ***F. verticillioides* (morphological and PCR based-Tef1α)** | **PQ468707** |
| 25/1F | Tápiószentmárton | Pest | Maize (Hungary) | *Fusarium* sp. (morphological) |  |
| 47/1F | Vértesacsa | Fejér | Maize (Hungary) | *Fusarium* sp. (morphological) |  |
| **46/1F** | **Enying** | **Fejér** | **Maize (Hungary)** | ***F. subglutinans* (morphological and PCR based- Tef1α)** | **PQ468708** |
| **56/1F** | **Csány** | **Heves** | **Maize (Hungary)** | ***F. verticillioides* (morphological and PCR based-Tef1α)** | **PQ468709** |
| 13/3F | Nyáregyháza | Pest | Maize (Hungary) | *Fusarium* sp. (morphological) |  |
| 12/1F | Pest megye county | Pest | Maize (Hungary) | *Fusarium* sp. (morphological) |  |
| **10/1F** | **Hajdú-Bihar county** | **Hajdú-Bihar** | **Maize (Hungary)** | **F. verticillioides (morphological and PCR based-Tef1α)** | **PQ468711** |
| **6/1F** | **Ceglédbercel** | **Pest** | **Maize (Hungary)** | ***F. verticillioides* (morphological and PCR based-Tef1α)** | **PQ468712** |
| **2/2F** | **Ceglédbercel** | **Pest** | **Maize (Hungary)** | ***F. verticillioides* (morphological and PCR based-Tef1α)** | **PQ468713** |
| **1/1F** | **Mezőkeresztes** | **Borsod-Abaúj-Zemplén** | **Maize (Hungary)** | ***F. verticillioides* (morphological and PCR based-Tef1α)** | **PQ468714** |
| 44/2F | Kunsziget | Győr-Moson-Sopron | Maize (Hungary) | *Fusarium* sp. (morphological) |  |
| 43/1F | Kunsziget | Győr-Moson-Sopron | Maize (Hungary) | *Fusarium* sp. (morphological) |  |
| 28/1F | Tura | Pest | Maize (Hungary) | *Fusarium* sp. (morphological) |  |
| **22/2F** | **Budapest** | **Pest** | **Maize (Hungary)** | ***F. verticillioides* (morphological and PCR based-Tef1α)** | **PQ468715** |
| 42/1F | Kisláng | Fejér | Maize (Hungary) | *Fusarium* sp. (morphological) |  |
| 32/2F | Kerta | Veszprém | Maize (Hungary) | *Fusarium* sp. (morphological) |  |
| 41/2F | Alsómocsolád | Baranya | Maize (Hungary) | *Fusarium* sp. (morphological) |  |
| 22/3F | Budapest | Pest | Maize (Hungary) | *Fusarium* sp. (morphological) |  |
| **53/1F** | **Agárd** | **Fejér** | **Maize (Hungary)** | ***F. verticillioides* (morphological and PCR based-Tef1α)** | **PQ468716** |
| 40/2F | Nagykáta | Pest | Maize (Hungary) | *Fusarium* sp. (morphological) |  |
| 44/1F | Kunsziget | Győr-Moson-Sopron | Maize (Hungary) | *Fusarium* sp. (morphological) |  |
| 36/1F | Pócsmegyer | Pest | Maize (Hungary) | *Fusarium* sp. (morphological) |  |
| 50/2F | Tök | Pest | Maize (Hungary) | *Fusarium* sp. (morphological) |  |
| 53/2F | Agárd | Fejér | Maize (Hungary) | *Fusarium* sp. (morphological) |  |
| 15/2F | Pest megye | Pest | Maize (Hungary) | *Fusarium* sp. (morphological) |  |
| 3/2F | Pest megye | Pest | Maize (Hungary) | *Fusarium* sp. (morphological) |  |
| 27/1F | Adásztevel | Veszprém | Maize (Hungary) | *Fusarium* sp. (morphological) |  |
| 39/1F | Tököl | Pest | Maize (Hungary) | *Fusarium* sp. (morphological) |  |
| 16/1F | Paks | Tolna | Maize (Hungary) | *Fusarium* sp. (morphological) |  |
| 9/1F | Albertirsa | Pest | Maize (Hungary) | *Fusarium* sp. (morphological) |  |
| 26/2F | Pilis | Pest | Maize (Hungary) | *Fusarium* sp. (morphological) |  |
| 51/1F | Bakonycsernye | Fejér | Maize (Hungary) | *Fusarium* sp. (morphological) |  |
| **13/3F** | **Nyáregyháza** | **Pest** | **Maize (Hungary)** | ***F. verticillioides* (morphological and PCR based-Tef1α)** | **PQ468710** |
| 9/2F | Albertirsa | Pest | Maize (Hungary) | *Fusarium* sp. (morphological) |  |
| **4/2F** | **Albertirsa** | **Pest** | **Maize (Hungary)** | ***F. proliferatum* (morphological and PCR based-Tef1α)** | **PQ468717** |
| **12/3F** | **Pest county** | **Pest** | **Maize (Hungary)** | ***F. verticillioides* (morphological and PCR based-Tef1α)** | **PQ468718** |
| 34/1F | Ráckeresztúr | Fejér | Maize (Hungary) | *Fusarium* sp. (morphological) |  |
| 28/2F | Tura | Pest | Maize (Hungary) | *Fusarium* sp. (morphological) |  |
| 46/2F | Enying | Fejér | Maize (Hungary) | *Fusarium* sp. (morphological) |  |
| 2892 | Keszthely | Zala | Maize (Hungary) | *Fusarium* sp. (morphological) |  |
| 2893 | Sárhatvan | Fejér | Maize (Hungary) | *Fusarium* sp. (morphological) |  |
| 3045 | Vácszentlászló | Pest | Maize (Hungary) | *Fusarium* sp. (morphological) |  |
| 9/3F | Albertirsa | Pest | Maize (Hungary) | *Fusarium* sp. (morphological) |  |
| 10/2F | Hajdú-Bihar county | Hajdú-Bihar | Maize (Hungary) | *Fusarium* sp. (morphological) |  |
| **14/2F** | **Pest megye county** | **Pest** | **Maize (Hungary)** | ***F. proliferatum* (morphological and PCR based-Tef1α)** | **PQ468719** |
| **23/3F** | **Káva** | **Pest** | **Maize (Hungary)** | ***F. proliferatum* (morphological and PCR based-Tef1α)** | **PQ468720** |
| **23/5F** | **Káva** | **Pest** | **Maize (Hungary)** | ***F. proliferatum* (morphological and PCR based-Tef1α)** | **PQ468721** |
| 24/1F | Romhány | Nógrád | Maize (Hungary) | *Fusarium* sp. (morphological) |  |
| **24/3F** | **Romhány** | **Nógrád** | **Maize (Hungary)** | ***F. proliferatum* (morphological and PCR based-Tef1α)** | **PQ468722** |
| **41/1F** | **Alsómocsolád** | **Baranya** | **Maize (Hungary)** | ***F. verticillioides* (morphological and PCR based-Tef1α)** | **PQ468723** |
| 57/1F | Pálfa | Tolna | Maize (Hungary) | *Fusarium* sp. (morphological) |  |
| 50/1F | Tök | Pest | Maize (Hungary) | *Fusarium* sp. (morphological) |  |
| 11/1F | Pest megye county | Pest | Maize (Hungary) | *Fusarium* sp. (morphological) |  |
| 13/1F | Nyáregyháza | Pest | Maize (Hungary) | *Fusarium* sp. (morphological) |  |
| **22/1F** | **Budapest** | **Pest** | **Maize (Hungary)** | ***F. verticillioides* (morphological and PCR based-Tef1α)** | **PQ468724** |
| 55/1F | Pápateszér | Veszprém | Maize (Hungary) | *Fusarium* sp. (morphological) |  |
| 40/1F | Nagykáta | Pest | Maize (Hungary) | *Fusarium* sp. (morphological) |  |
| 11/2F | Pest megye | Pest | Maize (Hungary) | *Fusarium* sp. (morphological) |  |
| 13/2F | Nyáregyháza | Pest | Maize (Hungary) | *Fusarium* sp. (morphological) |  |
| 21/2F | Csongrád | Csongrád-Csanád | Maize (Hungary) | *Fusarium* sp. (morphological) |  |
| 23/2F | Káva | Pest | Maize (Hungary) | *Fusarium* sp. (morphological) |  |
| 24/2F | Romhány | Nógrád | Maize (Hungary) | *Fusarium* sp. (morphological) |  |
| 24/4F | Romhány | Nógrád | Maize (Hungary) | *Fusarium* sp. (morphological) |  |
| 24/5F | Romhány | Nógrád | Maize (Hungary) | *Fusarium* sp. (morphological) |  |
| 59/1F | Érsekvadkert | Nógrád | Maize (Hungary) | *Fusarium* sp. (morphological) |  |
| 60/3F | Hernád | Pest | Maize (Hungary) | *Fusarium* sp. (morphological) |  |
| 5/1F | Dánszentmiklós | Pest | Maize (Hungary) | *Fusarium* sp. (morphological) |  |
| 14/1F | Pest megye | Pest | Maize (Hungary) | *Fusarium* sp. (morphological) |  |
| 45/1F | Felgyő | Csongrád | Maize (Hungary) | *Fusarium* sp. (morphological) |  |
| 45/2F | Felgyő | Csongrád | Maize (Hungary) | *Fusarium* sp. (morphological) |  |
| 50/2F | Tök | Pest | Maize (Hungary) | *Fusarium* sp. (morphological) |  |
| **39/2F** | **Tököl** | **Pest** | **Maize (Hungary)** | ***F. proliferatum* (morphological and PCR based-Tef1α)** | **PQ468725** |
| **37/2F** | **Solt** | **Bács-Kiskun** | **Maize (Hungary)** | ***F. verticillioides* (morphological and PCR based-Tef1α)** | **PQ468726** |
| 40/3F | Nagykáta | Pest | Maize (Hungary) | *Fusarium* sp. (morphological) |  |
| 48/1F | Sződ | Pest | Maize (Hungary) | *Fusarium* sp. (morphological) |  |
| 49/1F | Kiskunfélegyháza | Bács-Kiskun | Maize (Hungary) | *Fusarium* sp. (morphological) |  |
| 52/1F | Kajárpéc | Győr-Moson-Sopron | Maize (Hungary) | *Fusarium* sp. (morphological) |  |
| 2/1F | Ceglédbercel | Pest | Maize (Hungary) | *Fusarium* sp. (morphological) |  |
| 23/4F | Káva | Pest | Maize (Hungary) | *Fusarium* sp. (morphological) |  |
