# Supplementary material for: Co-Occurrence of Beauvericin and Fumonisin Producing Ability of Fusarium Strains Isolated from Crop Plants in Hungary
Source: Curr Microbiol. 2025 May 23;82(7):302. doi: 10.1007/s00284-025-04243-9 (PMC12101999; doi:10.1007/s00284-025-04243-9)
Supplement: Supplementary file 4 — Supplementary file4 (DOCX 22 KB) [file 284_2025_4243_MOESM4_ESM.docx]

Article title: Co-occurrence of beauvericin and fumonisin producing ability of Fusarium strains isolated from crop plants in Hungary

Journal name: Current Microbiology

Authors: Ákos Suhajda, Mohammed Al-Nussairawi, Ines Amara, Csilla Sörös, Rita Tömösközi-Farkas, Balázs Kriszt, Milán Farkas, Mátyás Cserháti

Corresponding author: Balázs Kriszt, [Kriszt.Balázs@uni-mate.hu](mailto:Kriszt.Balázs@uni-mate.hu)

| **A comprehensive overview of the extant literature to BEA production experiments.** | | | | | | | |
| --- | --- | --- | --- | --- | --- | --- | --- |
| *Fusarium isolate* | BEA production (ppm) | Matrix | Moisture | Spore cc. | Temperature | Incubation time | Reference |
| ***F. verticilloides* (0/164)** | **ND** | Maize (100 g) | 45% moisture | 1ml x 10⁷ | 25°C | 4 week in darkness | DOI 10.1007/s12550-011-0095-6 |
| *F. proliferatum* (6/15) | 15-43 ppm (mean) | Maize (100 g) | 45% moisture | 1ml x 10⁷ | 25°C | 4 week in darkness |  |
| *F. subglutinans* (5/40) | 3.5-12.9 ppm (mean) | Maize (100 g) | 45% moisture | 1ml x 10⁷ | 25°C | 4 week in darkness |  |
| *F. sacchari* (46/46) | all positive (TLC test) | Maize (15 g) | 45% moisture | 1ml x 10⁶ | room temperature | 28 days in darkness | DOI 10.1590/1678-4324-2021200088 |
| *F. subglutinans* (9/9) | all postive (TLC test) | Maize (15 g) | 45% moisture | 1ml x 10⁶ | room temperature | 28 days in darkness |  |
| ***F. verticilloides* (0/7)** | **ND** | Rice (30 g) | 13 mL water | 5 plugs of 0.6 cm in diameter of each culture | 25°C | 21 days in darkness | DOI 10.1016/j.ijfoodmicro.2020.108667 |
| *F. proliferatum* (8/8) | 244-858 ppm | Rice (30 g) | 13 mL water |  | 25°C | 21 days in darkness |  |
| *F. fujikuroi* (7/8) | 55-1501 ppm | Rice (30 g) | 13 mL water |  | 25°C | 21 days in darkness |  |
| *F. volatile* (7/7) | 68-3467 ppm | Rice (30 g) | 13 mL water |  | 25°C | 21 days in darkness |  |
| *F. anthophilum* (5/6) | 68-1811 ppm | Rice (30 g) | 13 mL water |  | 25°C | 21 days in darkness |  |
| *F. pseudocircinatum* (2/2) | 685-1906 ppm | Rice (30 g) | 13 mL water |  | 25°C | 21 days in darkness |  |
| *F. sterilihyphosum* (2/2) | 39-44 ppm | Rice (30 g) | 13 mL water |  | 25°C | 21 days in darkness |  |
| *F. begoniae* (1/1) | 54 ppm | Rice (30 g) | 13 mL water |  | 25°C | 21 days in darkness |  |
| *F. culmorum* (0/8) | ND | Maize (200 g) | 45% moisture | 2ml x 10⁷ | 25°C | 4 week in darkness | DOI 10.1128/AEM.64.8.3084-3088.1998. |
| *F. cerealis* (0/5) | ND | Maize (200 g) | 45% moisture | 2ml x 10⁷ | 25°C | 4 week in darkness |  |
| *F. graminearum* (0/5) | ND | Maize (200 g) | 45% moisture | 2ml x 10⁷ | 25°C | 4 week in darkness |  |
| *F. sambucinum* (12/14) | 2-230 ppm | Maize (200 g) | 45% moisture | 2ml x 10⁷ | 25°C | 4 week in darkness |  |
| *F. veneatum* (0/3) | ND | Maize (200 g) | 45% moisture | 2ml x 10⁷ | 25°C | 4 week in darkness |  |
| *F. torulosum* (0/7) | ND | Maize (200 g) | 45% moisture | 2ml x 10⁷ | 25°C | 4 week in darkness |  |
| *F. flocciferum* (0/2) | ND | Maize (200 g) | 45% moisture | 2ml x 10⁷ | 25°C | 4 week in darkness |  |
| *F. acuminatum var. acuminatum* (1/4) | 8 ppm | Maize (200 g) | 45% moisture | 2ml x 10⁷ | 25°C | 4 week in darkness |  |
| *F. acuminatum var. armeniacum* (1/3) | 2 ppm | Maize (200 g) | 45% moisture | 2ml x 10⁷ | 25°C | 4 week in darkness |  |
| *F. compactum* (0/3) | ND | Maize (200 g) | 45% moisture | 2ml x 10⁷ | 25°C | 4 week in darkness |  |
| *F. scirpi* (0/1) | ND | Maize (200 g) | 45% moisture | 2ml x 10⁷ | 25°C | 4 week in darkness |  |
| *F. equiseti* (2/3) | 3-12 ppm | Maize (200 g) | 45% moisture | 2ml x 10⁷ | 25°C | 4 week in darkness |  |
| *F. longipes* (1/2) | 200 ppm | Maize (200 g) | 45% moisture | 2ml x 10⁷ | 25°C | 4 week in darkness |  |
| *F. subglutinans* (3/3) | 10-300 ppm | Maize (200 g) | 45% moisture | 2ml x 10⁷ | 25°C | 4 week in darkness |  |
| *F. anthophilum* (1/2) | 1200 ppm | Maize (200 g) | 45% moisture | 2ml x 10⁷ | 25°C | 4 week in darkness |  |
| *F. oxysporum* (3/7) | 13-3200 ppm | Maize (200 g) | 45% moisture | 2ml x 10⁷ | 25°C | 4 week in darkness |  |
| *F. chlamydosporum* (0/1) | ND | Maize (200 g) | 45% moisture | 2ml x 10⁷ | 25°C | 4 week in darkness |  |
| *F. poae* (3/4) | 20-63 ppm | Maize (200 g) | 45% moisture | 2ml x 10⁷ | 25°C | 4 week in darkness |  |
| *F. sporotrichioides* (0/2) | ND | Maize (200 g) | 45% moisture | 2ml x 10⁷ | 25°C | 4 week in darkness |  |
| *F. tricinctum* (0/2) | ND | Maize (200 g) | 45% moisture | 2ml x 10⁷ | 25°C | 4 week in darkness |  |
| *F. avenaceum* (1/6) | 7 ppm | Maize (200 g) | 45% moisture | 2ml x 10⁷ | 25°C | 4 week in darkness |  |
| *F. polyphialidicum* (0/1) | ND | Maize (200 g) | 45% moisture | 2ml x 10⁷ | 25°C | 4 week in darkness |  |
| *F. beomiforme* (1/1) | 5 ppm | Maize (200 g) | 45% moisture | 2ml x 10⁷ | 25°C | 4 week in darkness |  |
| *F. nygamai* (2/2) | 3-19 ppm | Maize (200 g) | 45% moisture | 2ml x 10⁷ | 25°C | 4 week in darkness |  |
| *F. dlamini* (2/2) | 19-94 ppm | Maize (200 g) | 45% moisture | 2ml x 10⁷ | 25°C | 4 week in darkness |  |
| ***F. verticilloides* (0/5)** | **ND** | Maize (400 g) | Water (400 mL) | 2ml x 10⁷ | 25°C | 21 days in darkness | DOI 10.1021/jf9903713 |
| *F. proliferatum* (4/5) | 10-1725 ppm | Maize (400 g) | Water (400 mL) | 2ml x 10⁷ | 25°C | 21 days in darkness |  |
| *F. subglutinans* (4/5) | 330-2630 ppm | Maize (400 g) | Water (400 mL) | 2ml x 10⁷ | 25°C | 21 days in darkness |  |
| *F. globosum* (1/5) | 25 ppm | Maize (400 g) | Water (400 mL) | 2ml x 10⁷ | 25°C | 21 days in darkness |  |
| ***F. verticillioides* (0/4)** | **ND** | Rice (10 g) | 45% moisture | 2ml x 10⁷ | 25°C | 3 weeks in darkness | DOI 10.1016/j.ijfoodmicro.2007.07.004 |
| *F. subglutinans* (0/3) | ND | Rice (10 g) | 45% moisture | 2ml x 10⁷ | 25°C | 3 weeks in darkness |  |
| *F. proliferatum* (7/7) | 5-1300 ppm | Rice (10 g) | 45% moisture | 2ml x 10⁷ | 25°C | 3 weeks in darkness |  |
| *F. acutatum* (5/5) | 200-350 ppm | Rice (10 g) | 45% moisture | 2ml x 10⁷ | 25°C | 3 weeks in darkness |  |
| *F. annulatum (1/1)* | ND | Rice (10 g) | 45% moisture | 2ml x 10⁷ | 25°C | 3 weeks in darkness |  |
| *F. anthophilum* (2/3) | 14-170 ppm | Rice (10 g) | 45% moisture | 2ml x 10⁷ | 25°C | 3 weeks in darkness |  |
| *F. begoniae* (0/2) | ND | Rice (10 g) | 45% moisture | 2ml x 10⁷ | 25°C | 3 weeks in darkness |  |
| *F. brevicatenulatum* (0/2) | ND | Rice (10 g) | 45% moisture | 2ml x 10⁷ | 25°C | 3 weeks in darkness |  |
| *F. bulbicola* (1/3) | 310 ppm | Rice (10 g) | 45% moisture | 2ml x 10⁷ | 25°C | 3 weeks in darkness |  |
| *F. circinatum* (3/4) | 59-120 ppm | Rice (10 g) | 45% moisture | 2ml x 10⁷ | 25°C | 3 weeks in darkness |  |
| *F. concentricum* (6/6) | 170-2700 ppm | Rice (10 g) | 45% moisture | 2ml x 10⁷ | 25°C | 3 weeks in darkness |  |
| *F. denticulatum* (3/5) | 7-3000 ppm | Rice (10 g) | 45% moisture | 2ml x 10⁷ | 25°C | 3 weeks in darkness |  |
| *F. dlamini* (3/4) | 2-20 ppm | Rice (10 g) | 45% moisture | 2ml x 10⁷ | 25°C | 3 weeks in darkness |  |
| *F. fujikuroi* (2/2) | 140-1500 ppm | Rice (10 g) | 45% moisture | 2ml x 10⁷ | 25°C | 3 weeks in darkness |  |
| F. globosum (0/3) | ND | Rice (10 g) | 45% moisture | 2ml x 10⁷ | 25°C | 3 weeks in darkness |  |
| *F. guttiforme* (3/5) | 5-540 ppm | Rice (10 g) | 45% moisture | 2ml x 10⁷ | 25°C | 3 weeks in darkness |  |
| *F. lactis* (1/2) | 190 ppm | Rice (10 g) | 45% moisture | 2ml x 10⁷ | 25°C | 3 weeks in darkness |  |
| *F. napiforme* (0/3) | ND | Rice (10 g) | 45% moisture | 2ml x 10⁷ | 25°C | 3 weeks in darkness |  |
| *F. nygamai* (2/3) | 13-1100 ppm | Rice (10 g) | 45% moisture | 2ml x 10⁷ | 25°C | 3 weeks in darkness |  |
| *F. phyllophilum* (2/2) | 13-340 ppm | Rice (10 g) | 45% moisture | 2ml x 10⁷ | 25°C | 3 weeks in darkness |  |
| *F. proliferatum* (10/10) | 5-1300 ppm | Rice (10 g) | 45% moisture | 2ml x 10⁷ | 25°C | 3 weeks in darkness |  |
| F. pseudoanthophilum (0/3) | ND | Rice (10 g) | 45% moisture | 2ml x 10⁷ | 25°C | 3 weeks in darkness |  |
| *F. pseudocircinatum* (2/3) | 90-200 ppm | Rice (10 g) | 45% moisture | 2ml x 10⁷ | 25°C | 3 weeks in darkness |  |
| F. pseudonygamai (0/2) | ND | Rice (10 g) | 45% moisture | 2ml x 10⁷ | 25°C | 3 weeks in darkness |  |
| *F. ramigenum* (0/2) | ND | Rice (10 g) | 45% moisture | 2ml x 10⁷ | 25°C | 3 weeks in darkness |  |
| *F. sacchari* (3/4) | 47-1400 ppm | Rice (10 g) | 45% moisture | 2ml x 10⁷ | 25°C | 3 weeks in darkness |  |
| *F. succisae* (1/2) | 330 ppm | Rice (10 g) | 45% moisture | 2ml x 10⁷ | 25°C | 3 weeks in darkness |  |
| *F. thapsinum* (0/4) | ND | Rice (10 g) | 45% moisture | 2ml x 10⁷ | 25°C | 3 weeks in darkness |  |
| *F. udum* (0/4) | ND | Rice (10 g) | 45% moisture | 2ml x 10⁷ | 25°C | 3 weeks in darkness |  |
